# New experimental model for single liver lobe hyperthermia in small animals using non-directional microwaves

**DOI:** 10.1371/journal.pone.0184810

**Published:** 2017-09-21

**Authors:** Ionuț Tudorancea, Vlad Porumb, Alexandru Trandabăţ, Decebal Neaga, Bogdan Tamba, Radu Iliescu, Gabriel M. Dimofte

**Affiliations:** 1 Department of Physiology, Grigore T. Popa University of Medicine and Pharmacy, Iasi, Romania; 2 Department of Surgery, Grigore T. Popa University of Medicine and Pharmacy, Iasi, Romania; 3 Department of Surgery, Regional Institute of Oncology, Iasi, Romania; 4 Faculty of Electrical Engineering, Gheorghe Asachi Technical University, Iaşi, Romania; 5 Department of Engineering, Regional Institute of Oncology, Iasi, Romania; 6 Department of Pharmacology, Grigore T. Popa University of Medicine and Pharmacy, Iasi, Romania; University of Florida, UNITED STATES

## Abstract

**Purpose:**

Our aim was to develop a new experimental model for *in vivo* hyperthermia using non-directional microwaves, applicable to small experimental animals. We present an affordable approach for targeted microwave heat delivery to an isolated liver lobe in rat, which allows rapid, precise and stable tissue temperature control.

**Materials and methods:**

A new experimental model is proposed. We used a commercial available magnetron generating 2450 MHz, with 4.4V and 14A in the filament and 4500V anodic voltage. Modifications were required in order to adjust tissue heating such as to prevent overheating and to allow for fine adjustments according to real-time target temperature. The heating is controlled using a virtual instrument application implemented in LabView® and responds to 0.1° C variations in the target. Ten healthy adult male Wistar rats, weighing 250–270 g were used in this study. The middle liver lobe was the target for controlled heating, while the rest of the living animal was protected.

**Results:**

In vivo microwave delivery using our experimental setting is safe for the animals. Target tissue temperature rises from 30°C to 40°C with 3.375°C / second (R^2^ = 0.9551), while the increment is lower it the next two intervals (40–42°C and 42–44°C) with 0.291°C/ s (R^2^ = 0.9337) and 0.136°C/ s (R^2^ = 0.7894) respectively, when testing in sequences. After reaching the desired temperature, controlled microwave delivery insures a very stable temperature during the experiments.

**Conclusions:**

We have developed an inexpensive and easy to manufacture system for targeted hyperthermia using non-directional microwave radiation. This system allows for fine and stable temperature adjustments within the target tissue and is ideal for experimental models testing below or above threshold hyperthermia

## Introduction

Numerous experiments have been undertaken using heat in cancer therapies, with a wide range of delivery methods, generating a local, regional or whole-body hyperthermia [1–10]. As compared with normal tissues, cancer bearing tissues have been shown to be more susceptible to death in hyperthermia [11,12] and experimental treatment strategies have used thermal manipulation of tumors either by nonspecific tissue disruption, or as an adjuvant to chemotherapy in local or systemic delivery [11,13–17]. Heat induced alterations in target tissue depend on the temperature achieved and the duration of the exposure. Applying heat over 45°C quickly induces necrosis, due to protein coagulation [18–20]. However, tissue exposed to temperatures below 43–45°C displays minimal and even reversible changes, usually without structural alterations visible in light microscopy, but the threshold for subliminal changes is variable and depends on the model used and the tissue exposed to hyperthermia [20–25]. Nevertheless, the failure of the early approaches using direct heat-induced cell killing has prompted interest in the biological effects of mild hyperthermia (39–42°C), especially in conjunction with other cancer therapies. Indeed, mild hyperthermia improves tumor oxygenation, inhibits damage repair while also potentially improving nanotechnology-based targeted delivery [26].

Exposure of three-dimensional tissue volumes to temperatures within the narrow interval of 42–45°C may induce variable effects, ranging from minimal functional alterations to necrosis, due to non-uniform spatial distribution of the temperature load, which is highly dependent on the modalities used for heat delivery [27–29]. All current methods for thermal manipulation fall short of the goal of achieving a uniform temperature load within target tissues volume. Consequently, a range of thermal responses will be generated within the tissue and temperature-specific effects will be difficult to discern. Therefore, in order to study the selective effect of hyperthermia, especially in subliminal conditions (42–45°C), tissue temperatures must be precisely controlled and tissue volume heating achieved in a uniform manner.

Our aim was to develop a new experimental model for *in vivo* mild hyperthermia using non-directional microwaves, applicable to small live animals. We present an affordable approach for targeted microwave heat delivery to an isolated liver lobe in rat, which allows rapid, precise and stable tissue temperature control, while completely avoiding extraneous heating of non-targeted tissues. This experimental model may be useful for the study of temperature–specific alterations in tissues exposed to a uniform temperature load, with potential applications in therapeutic strategies involving hyperthermia.

## Material and methods

### Microwave heating model

A modified commercial microwave oven was used in order to deliver non-directional microwaves to live tissue, which in our experiments was the middle liver lobe of the experimental animal. We used a commercial available 2M214-LG® (LG, Seoul, South Korea) magnetron generating 2450 MHz, with 4.4V and 14A in the filament and 4500V anodic voltage. Modifications were required in order to adjust tissue heating such as to prevent overheating and to allow for fine adjustments according to real-time target temperature.

**Fig 1:**
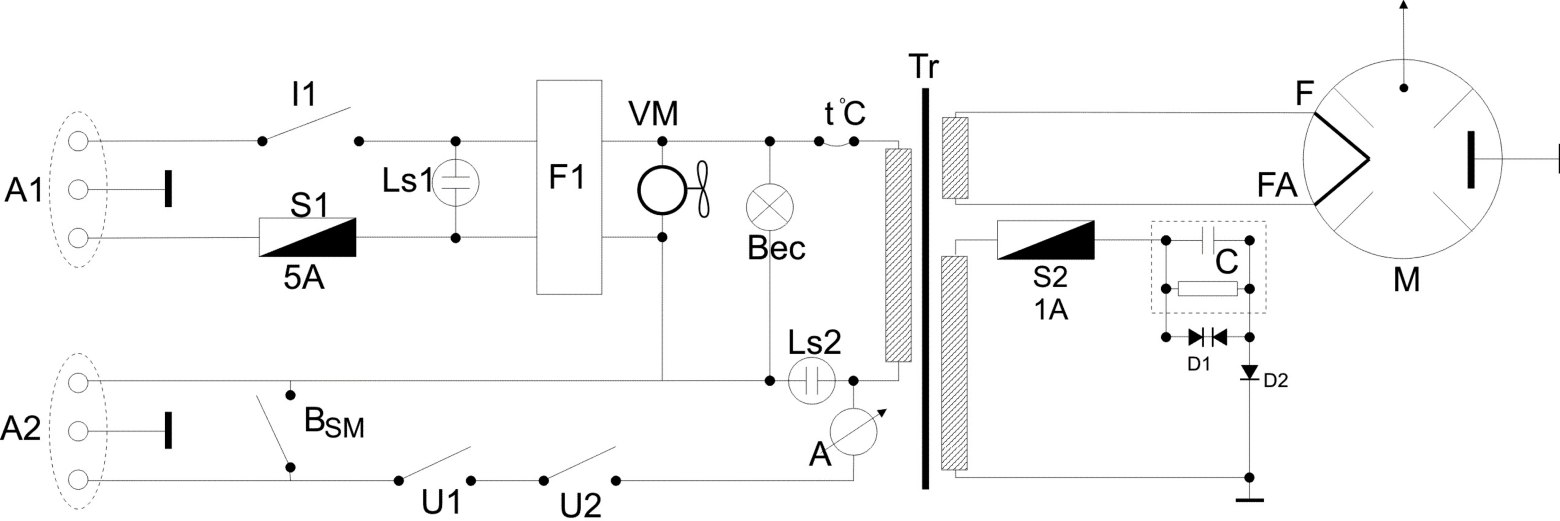
Wiring diagram of the magnetron. U1, U2 –dor close switch; A1, A2 –connectors; I1 –main breaker; Ls1, Ls2 –signaling lamps; Tr–transformer; S1, S2 –fuses; BSM–manual command button; A–ampermeter; F1 –power filter; C–capacitor; D1 –diode HVR-U62; D2 –diode HVR; VM–magnetron chiller; M–magnetron.

Electrical circuits were removed from the original microwave, leaving only the magnetron power supply, magnetron cooling system, chamber light circuit and safety circuits that protects against the accidental door opening. A new electronic controller was designed for the magnetron, designed to operate on a feed-back loop, connected to temperature sensors in target tissue (Fig 1).

A1 connector is used for power supply (230V/10A), while A2 connector is used for temperature-dependent control. The original configuration of the microwave oven magnetron generates excessive energy in form of microwaves (1000 W), unsuitable for fine thermal manipulation in small tissue volumes. Selective targeting of the middle lobe of the rat liver creates the problem of a relatively small absorptive volume (less than a cubic centimeter), thus we introduced an additional target in the form of a 100-ml water-filled microwave resistant plastic container. This additional target, much larger than the targeted tissue volume, will absorb (using proportional distribution) most of the energy generated by the magnetron and prevent uncontrolled, rapid heating of the rat liver lobe [30]. Experiments run without the additional microwave energy trap (water target) resulted in excessive, uncontrolled heating of the target organ. A small plastic tube was used to evacuate water vapors outside the microwave oven chamber. The desired temperature inside the tissue target was achieved using a feedback-controlled, rapid pulse technology to power the magnetron, thus allowing for fine adjustments of the total energy transferred to tissue. Computer controlled adjustment of the ratio between the on and off intervals, based on tissue temperature measurements, led to the adequate decrease of the time-integrated power output and maintain tissue temperature with high accuracy.

**Fig 2:**
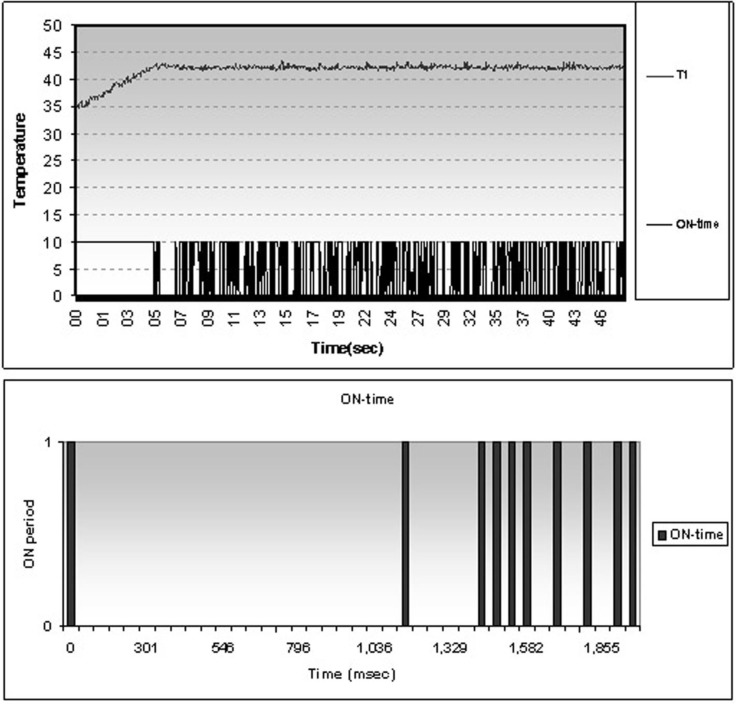
Magnetron duty cycle. Magnetron duty cycle in an experiment aiming to liver heating at 42°C (graph in the upper panel); On-off cycle during the first 2 seconds after reaching set temperature (graph in lower panel).

The sequence of magnetron activation is correlated with temperature readings in an experiment in order to increase the tissue temperature to 42° C (Fig 2).

When tissue temperature is below the established limit, the magnetron is powered continuously thus achieving a progressive, linear increase in target temperature. After reaching the set limit (42° C) the controller maintains the temperature using a rapid on-off switching, for a resulting magnetron duty cycle of 20.5% of time-period spent at the desired temperature.

Combining the additional water target and rapid pulsed activation of the magnetron we were achieved fine tuning of the amount of microwave energy delivered to target tissue, without the need of complex engineering required to change the power of the magnetron.

The revolving table was removed to allow for direct observation of live experimental animals, as well as for accurate positioning of the thermocouples. While we cannot exclude the possibility that the absence of a stirrer or rotating table generates a microwave pattern of interference inside the cavity, the presence of the energy absorbing water container and the reflecting surfaces in the random arrangement of the aluminum foil used to cover the body of the animal, minimized the likelihood of a hot spot affecting the small tissue sample exposed.

**Fig 3:**
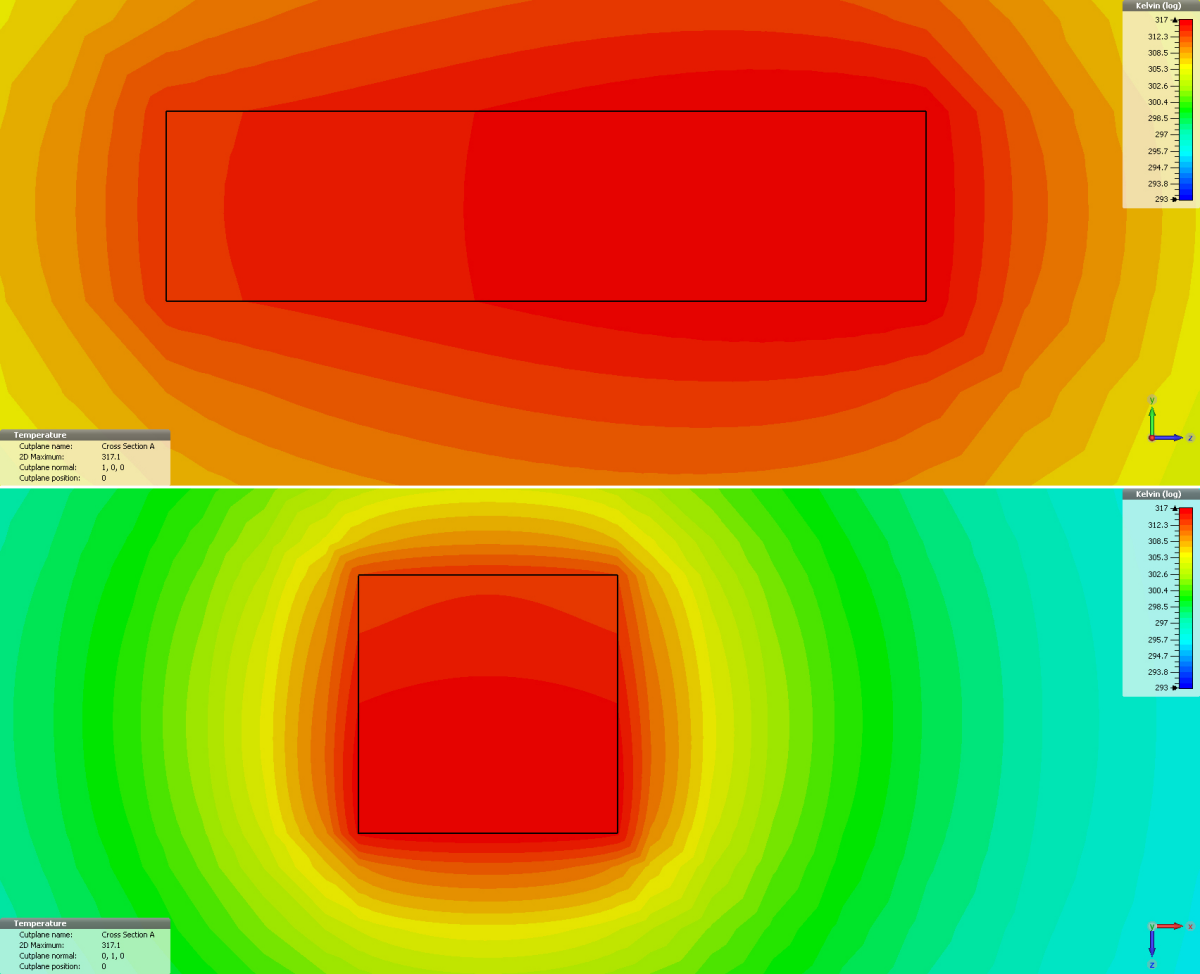
Electromagnetic/Thermal co-simulation. Electromagnetic/Thermal co-simulation using CST Studio Suit; vertical plane—upper panel; horizontal plane -lower panel.

Simulation of the thermal effect were performed using CST Studio Suite® (CST Darmstadt, Germany) using a predefined microwave chamber and source with parameters similar with those in the prototype. Thermal effect was simulated in a rectangular (2x2x0.5cm) tissue sample centrally placed in the oven using available parameters for relaxation and thermal characteristics of tissue samples (Foundation for Research on Information Technologies in Society, Zurich, Switzerland, http://www.itis.ethz.ch/itis-for-health/tissue-properties/downloads/; Institute for Applied Physics, Florence, Italy, http://niremf.ifac.cnr.it/, accessed on April 2016). Simulation performed with FDTD electromagnetic simulator at 2.45 GHz, central tissue temperature maintained at 40°C, wall temperature kept constant at 20°C and CST option "Bioheat"—not active. Computer simulation of the thermal effect predicts a homogenous heat distribution within the simulated tissue (Fig 3).

Temperature probes are placed centrally in the experimental middle liver lobe, approximately 1.5–2.0 mm from either border. Control temperature probes are placed in lateral liver lobes and peritoneal cavity. All metallic components were grounded, thus preventing microwave-induced electrical discharges in the capacitive field of the oven. Although we did not interfere with the oven’s operator safety structures, we evaluated microwave radiation, 5 cm in front of the oven door and around the oven body. Measured microwave energy was inferior to 1mW/cm, similar to a commercial microwave oven working in standard operation mode. As the heat dissipated from the water container was the only source of heating inside the cavity, a slight decrease in central body temperature of the experimental animal was observed (2–3 degrees).

Tissue temperature was monitored using highly sensitive J thermocouples, designed and produced especially for this experiment from 23G syringe needles. The sensitive junction was introduced into the hypodermic needle that acts as a shield. Signal wires were protected using a twisted shield cable [31]. The sensors shields (needle and shield cable) were connected to the system ground in order to avoid discharges in microwave field, as previously described [32–34].

Temperature data acquired from target tissue was used in order to maintain target temperature constant during the time-frame of the experiments. Thermal probes feed information into a virtual instrument (VI) application implemented in LabView® (National Instruments Corporation, Texas, USA). The temperature readings from the target tissue are analyzed in real time and the system responds to variations of 0.1° C, controlling the power of the magnetron by switching it automatically on/ off. For this purpose, we developed a virtual instrument (VI) that allows us to control the temperature in the liver lobe (S1 Fig).

**Fig 4:**
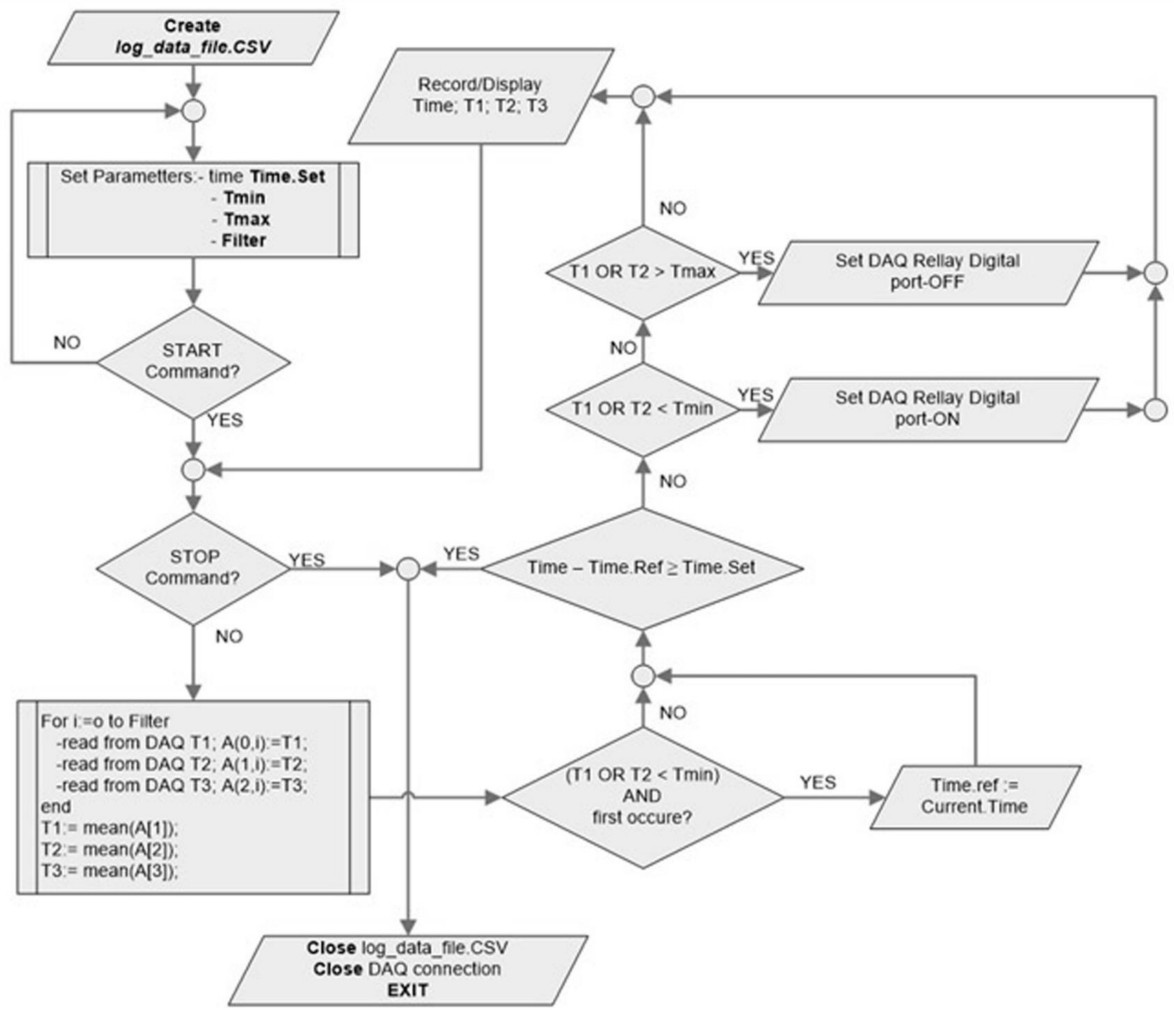
Virtual instrument programming module. Block diagram.

The VI software component aims to keep the target temperature within a set narrow interval for a given period of time. The VI contains two parts: the user interface and the programming module. The user interface (S2 Fig) is used during the experiment and allows the user to adjust main parameters: upper temperature limit, lower temperature limit, length of time, and filter accuracy. Heating time starts once the desired temperature is reached in T1 or T2 sensors. Once the instrument is running, the user can start the test by pressing the recording button, with real time temperature readings displayed in analogical and graphic modes. The programming module is the program behind the VI and the block diagram is presented in Fig 4.

The implemented algorithm is a controller that keeps at least one parameter value between set limits in imposed conditions (time). For that reason, we acquire analogical signals from J type thermocouples, through a data acquisition board. Data are compared with preset limits, and the relay controls the magnetron by switching it on or off, in order to maintain the desired temperature in target tissue.

The Virtual Instrument hierarchy highlights the dependency between program sub-VI, the dataflow between program sub-VI and one of its main advantages: modularity. Due to this modular implementation of the algorithm, the researchers can easy modify the functions or can add supplementary functions to main VI core. Therefore, the microwave heating virtual instrument is one versatile tool that can be easily adapted in order to conduct experiments with different test procedure requests.

Due to the high sensibility of the thermocouples (0.01°C), high amplification factor on DAQ settings, and high sampling rate (10 kHz), any discharge or any voltage artifact can induce false peaks on reading values. Therefore, we introduced in the main program a data filtering routine. The original temperature values sampled at 100 kHz are smoothed by a moving average window of 10 samples, and compared with set limits. The relay is switched on/off through a 5V DAQ digital port if measured average is outside or inside preset limitations. The system will activate the magnetron using a classical feed-back algorithm. Using a common grounding for magnetron, oven protective cage and all shields (animal shield and thermocouples shielding components) we manage to eliminate all spikes in our experiments, but we continue to use the filtering routine as an additional safety measure (S3 Fig).

### Animals

Ten healthy adult male Wistar rats, weighing 250–270 g were used in this study. The experimental animals were maintained in individually ventilated polyethylene cages with food and water *ad libitum*, under controlled ambient temperature (21 ± 0.5°C) and a 12h/12h light–dark cycle. All procedures were approved by the Ethical Committee of the Grigore T. Popa University of Medicine and Pharmacy, Iasi. Animals were clinically monitored daily throughout the study to ensure well-being and normal behavior.

### Surgery

Each rat was anaesthetized with a mixture of Ketamine (65 mg/kg) and Xylazine (15 mg/kg) administrated intraperitoneally. Before starting experiments, deep anesthesia was verified by pinching the hind paw or gently swabbing the cornea of the rat. Lack of any response was considered the confirmation of deep anesthesia. None of the animals required supplementation of anaesthesia during the experiment, and recovered within 60 minutes. During anesthesia and surgical procedure rats were placed on a heating surface in order to maintain the central temperature of the body around 37°C, as monitored by a rectal temperature probe.

Liver exposure was made after incision of the anterior wall on the *linea alba*, starting from the xiphoid process and extended 3 cm to the pubis. An optimal exposure of the middle liver lobe is necessary, and that is possible due to particular anatomy of rat liver. In this species, the middle liver lobe is rather isolated and mobile, especially after cutting the falciform ligament. We used in all experiments the middle lobe of the liver as a target tissue for microwave delivery, while the lateral lobes served as control.

**Fig 5:**
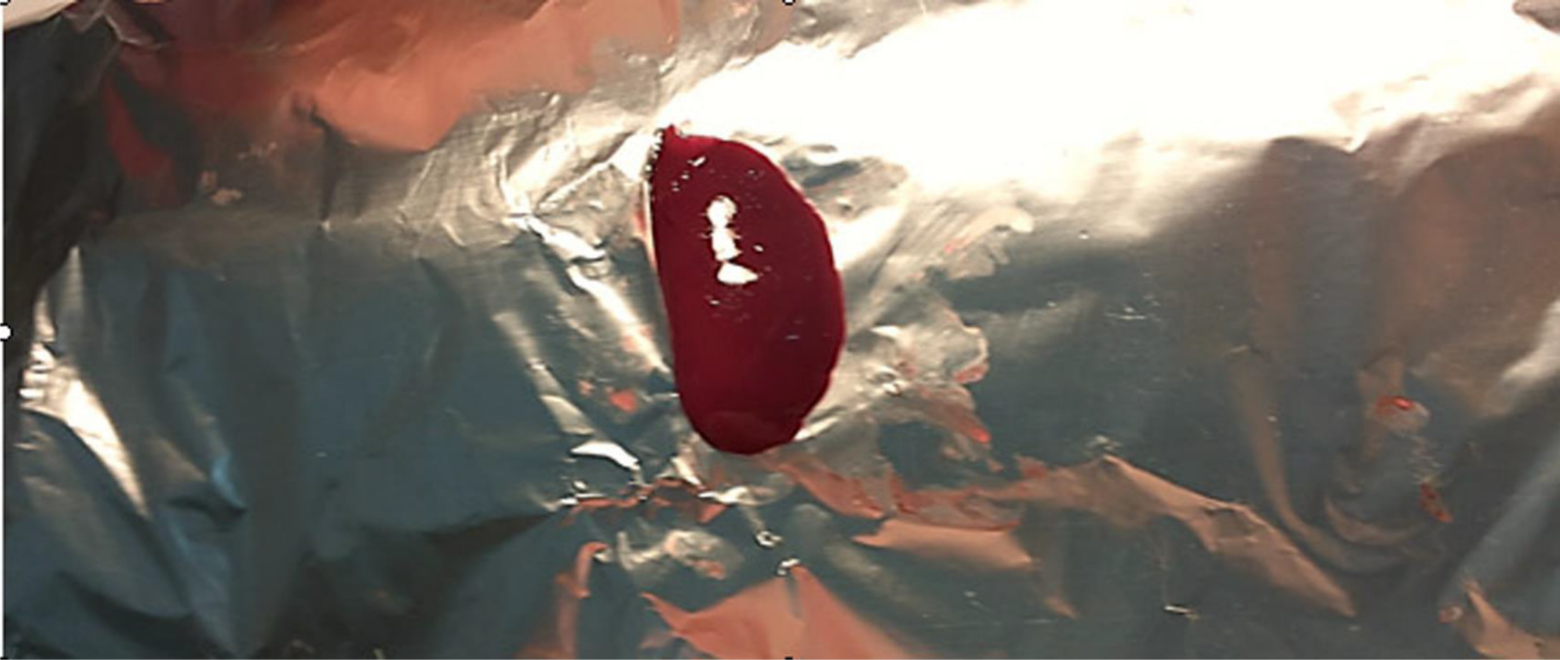
Aluminum foil wrapping. Mobilized middle liver lobe remains uncovered for microwave exposure.

The anaesthetized rat was protected from microwaves using aluminum foil that allows for a rapid and versatile wrapping (Fig 5). The whole body and non-target liver tissue were protected by this Faraday-type cage that was electrically connected to the oven protective cage and magnetron grounding electrode.

**Fig 6:**
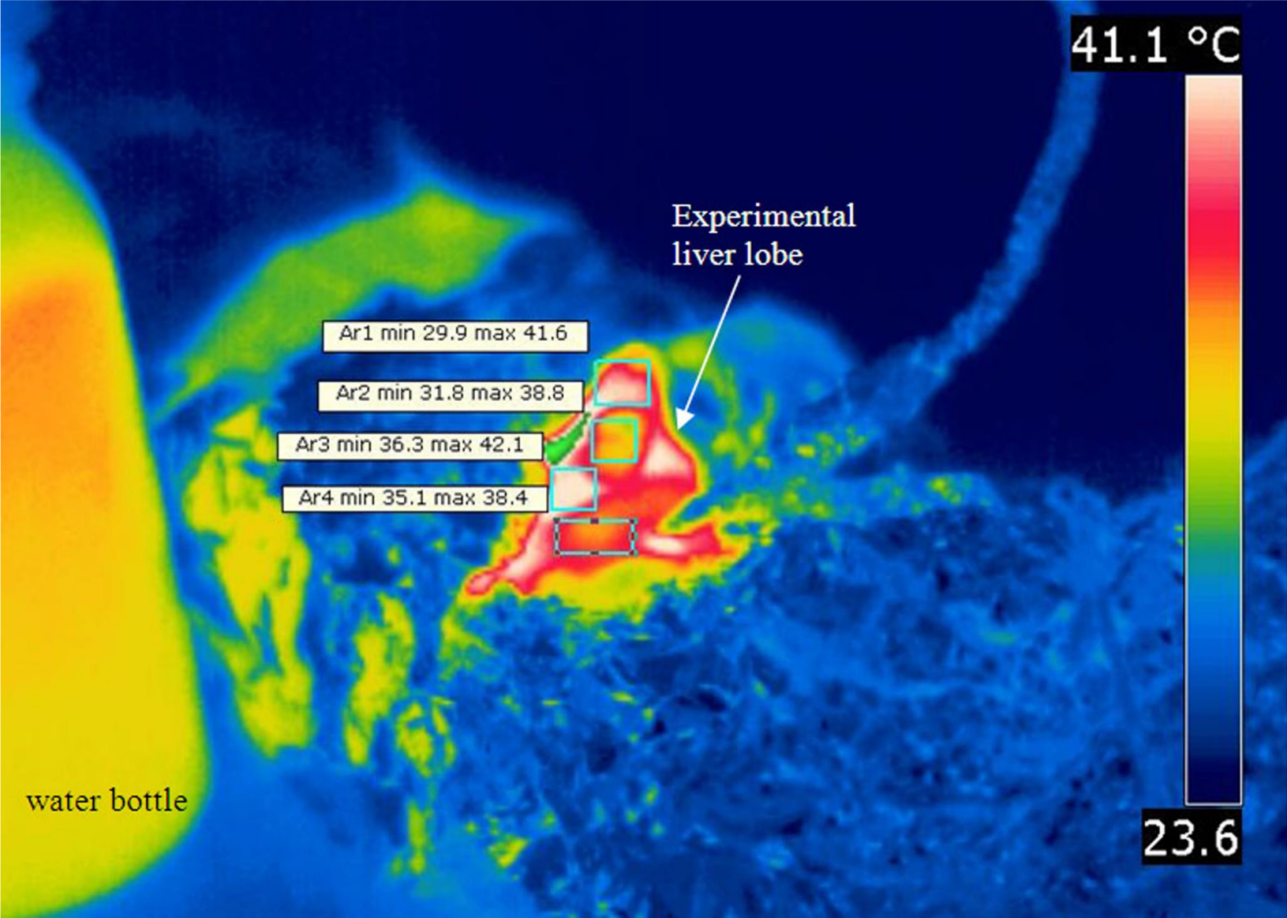
Thermal imaging after microwave exposure. Demonstration of the lack of hot spots outside experimental tissue. Infrared image obtained seconds after microwave heating—thermal gradient on the surface of the liver are inherent to rapid cooling in the periphery of the experimental lobe.

The efficiency of the protecting foil was assessed by two isolated temperature microprobes, one inserted into the abdominal cavity and the other one in the lateral liver lobe. A thermal image obtained immediately after targeted heating demonstrates that aluminum wrapping remains cold, while the liver lobe and water container are heated to a significantly higher temperature than the surrounding structures (Fig 6).

After the procedure (total time in microwave oven <3 minutes), the foil was removed, the micro sensors were detached and the abdomen was closed using non-absorbable suture silk. All animals survived the experimental procedure and were monitored during recovery and were brought back in special cages for postoperative follow-up. Meloxicam 1mg/kg was administered subcutaneously in the first 2 postoperative days, in order to ensure analgesia. No animals died or became ill before the experimental endpoint. Normal feeding was restored within 24 hours and animals were sacrificed by Xilazine/ Ketamine overdose followed by decapitation, 7 days after the procedure, for tissue evaluation in different experimental settings. For temperatures below 42°C there were no structural changes in light microscopy and no signs of burns associated with abnormal hot spots inside the experimental lobe.

## Results

**Fig 7:**
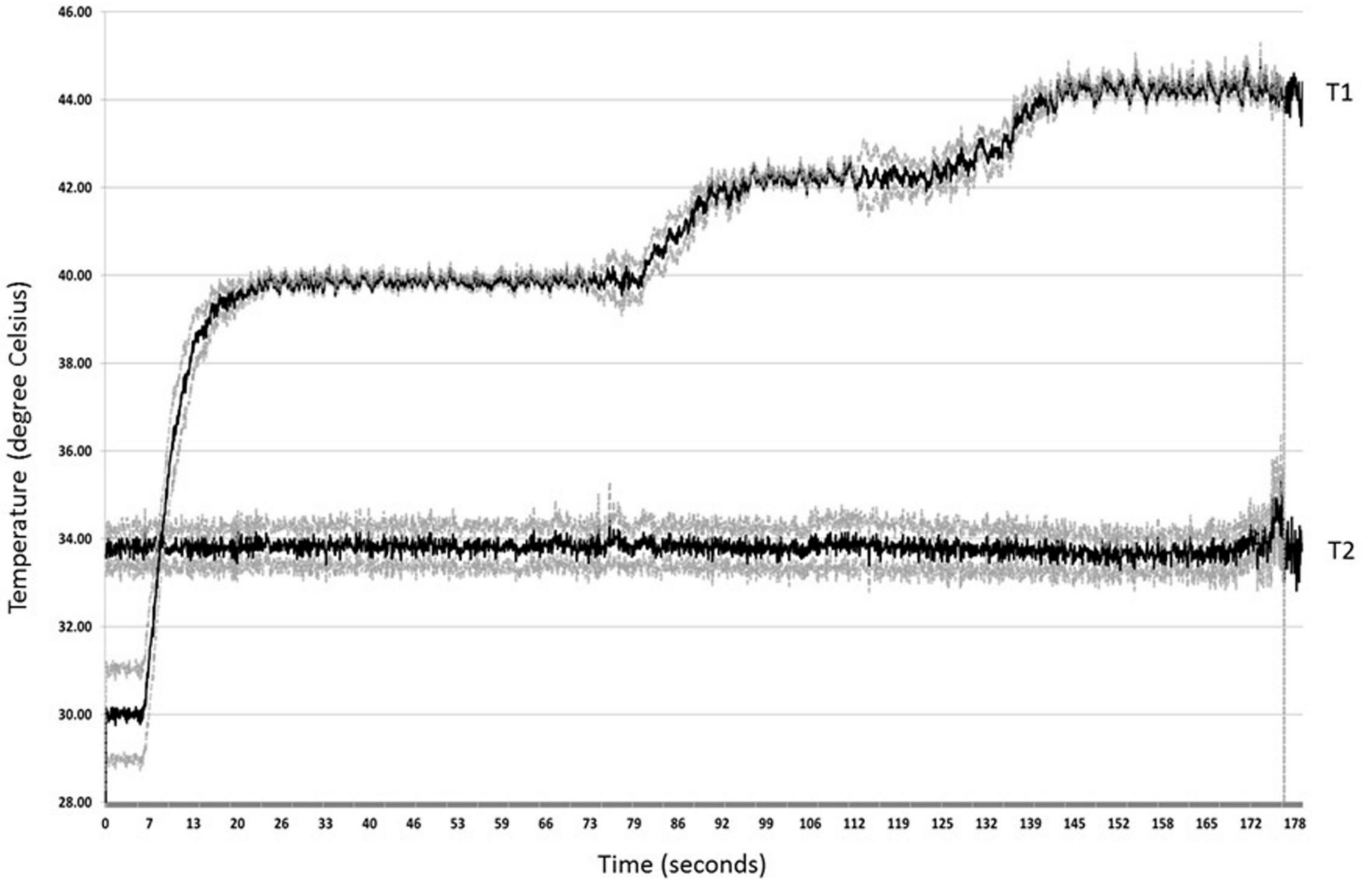
Continuous recording of tissue temperature. Temperature recorded for three successive targets (40, 42 and 44°C) (n = 10). T1—average of the temperatures values acquired from heated liver lobe. T2—average of the temperatures values acquired from control liver lobe. Dotted lines represent standard deviation.

Experimental data clearly demonstrates that the exposed middle lobe is being heated, while the rest of the animal is protected by the aluminum foil. After euthanasia, we harvested liver (heated and aluminum protected lobes), spleen, kidney and lungs. There were no changes associated with heat exposure that could be identified in light microscopy, as compared to normal rat histology. Just before heating, the temperature in exposed liver is somewhat lower than normal (Fig 7).

The 4°C difference at the beginning of the experiment is determined by the exposure to room temperature during surgical dissection and wrapping. The temperature in the experimental microwave chamber is also at room temperature before magnetron activation. Heat generated in the microwave chamber will not raise the central temperature of the rat as the aluminum protection acts as a very efficient screen. Two microprobes measured temperatures in the target tissue (T1) while two microprobes are positioned in the lateral liver lobe and the peritoneal cavity (T2). While desired temperature was maintained in the experimental lobe at set levels the rest of the body appears not to be affected, with very small variations around baseline. Fig 7 represents the plot for 10 experiments conducted in a similar manner, with target tissue heating set to 40°C, 42°C and 44°C in successive testing, with data presented as mean (black line) and standard deviation of the mean (dotted lines). We assume that successive increments in target temperature may be slightly biased due to tissue drying, but we minimized the effect by a rapid succession of intervals. All animals recovered after the experiment without any side effects, demonstrating the efficacy of the aluminum foil cage to protect the whole animal from the harmful effect of microwaves. In addition to the requirement for anesthesia, heating- induced dehydration of the target tissue limits the duration of the hyperthermic exposure in this experimental setup.

**Fig 8:**
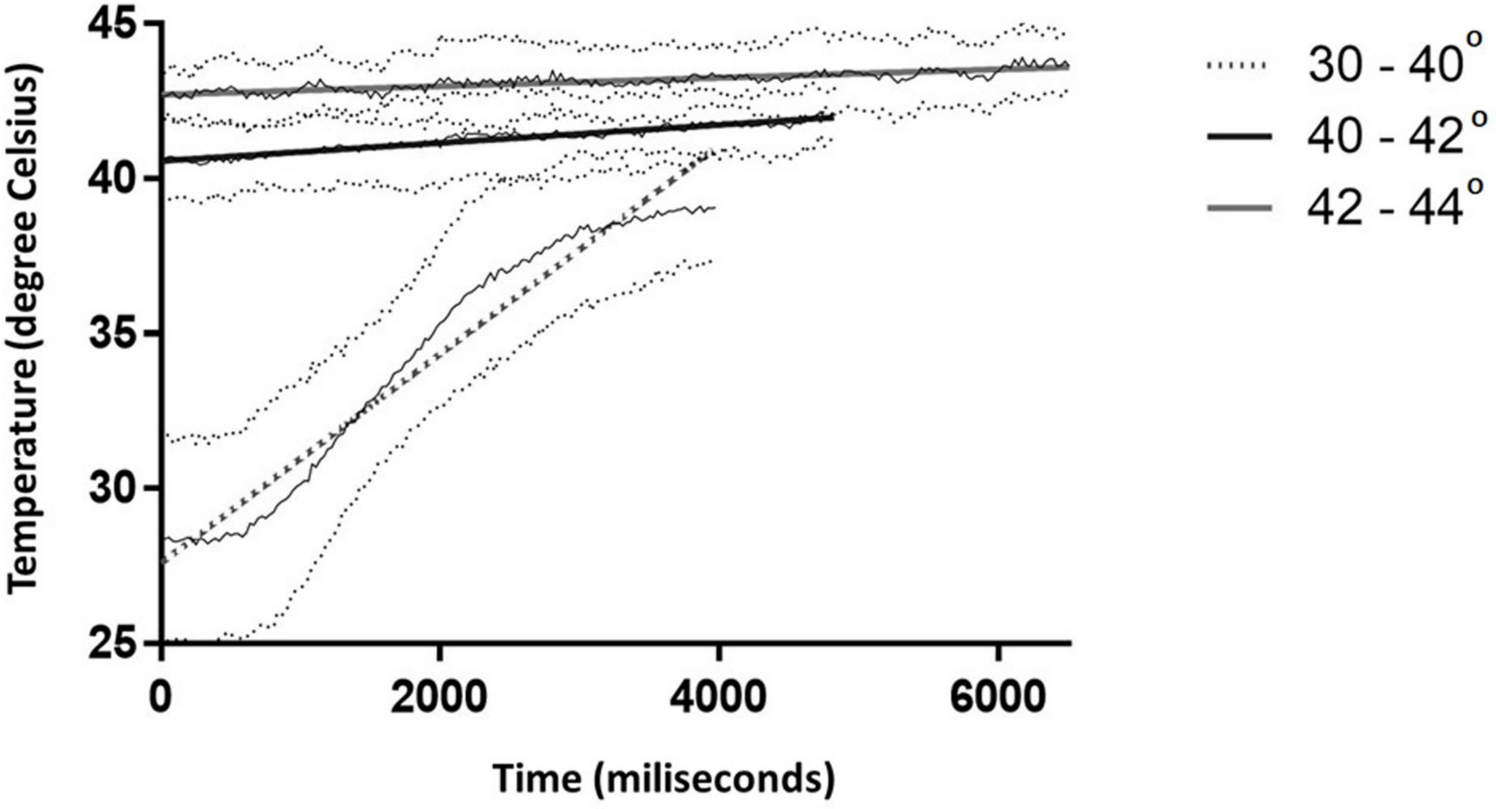
Linear regression indicating the rate of temperature increase in the heated lobe (n = 10). Thick dotted line: 30°C to 40°C at 3.375°C / second (R2 = 0.9551; p<0.0001); Thick black line: 40 to 42°C at 0.291°C/ second (R2 = 0.9337; p<0.0001); Thick gray line: 42 to 44°C at 0.136°C/ s (R2 = 0.7894; p<0.0001). Thin solid and dotted lines are average and respectively, standard deviation of recorded temperatures.

Increasing the target temperature from baseline to the desired level depends apparently on the temperature interval, which in turn is dependent on the feed-back algorithm. Larger thermal intervals imply a coarser microwave delivery, without frequent on-off switches that are needed to prevent overshooting. Temperature rises from 30°C to 40°C with 3.375°C / second (R^2^ = 0.9551), while the increment is lower it the next two intervals (40–42°C and 42–44°C) with 0.291°C/ s (R^2^ = 0.9337) and 0.136°C/ s (R^2^ = 0.7894) respectively, with very accurate linear regressions (Fig 8).

The differences in the slopes are generated by the temperature gradient required to achieve the set limit. A large temperature interval will allow for continuous activation of the magnetron and a steep slope, while a 2°C increment will generate short lived point temperatures within the set margins, as such turning the magnetron off and prolonging the time required to achieve a steady temperature. That is, in our view, an advantage of the system, that will prevent the rapid increase of temperature around the set limit and more precise heating of tissue volume.

## Discussions

Microwave induced heating has first been proposed for the treatment of recurrent breast cancer, and that opened the door to a wide series of applications using a range of thermal effects [35]. Microwaves generate heat at a rate which is proportional to the square of the applied electric field magnitude and the effective conductivity, a measurement of microwave absorption [36]. The precise mechanisms of interaction with tissue are very complex and difficult to predict, as energy delivery varies with radiating power, specific absorption rate, permittivity, depth of penetration and tissue conductivity.

Animal tissues contain ~ 70% water and thus will easily absorb microwave energy, producing a direct heating [37]. Widespread use of microwave energy for hyperthermic cancer therapy is limited due to rapid absorption in any water containing structures, unpredictable and rapid thermal changes, and deleterious effects to normal tissues. Targeted microwaves exposure would provide a conceptual advantage, although physical methods to focus microwave radiation are rather impractical in clinical or experimental settings. However, collimated microwave radiation would induce a significant thermal gradient within the targeted tissue along the direction of propagation, as effective microwave energy would decrease exponentially with the distance from the source. In contrast, a uniform distribution of the microwave radiation across the entire surface of the tissue, will allow a certain temperature to be achieved in the center, while the temperature gradient will be blunted as compared to that generated by directional microwaves.

We developed an experimental model for tissue hyperthermia designed to generate accurate mild heating of target tissue in vivo. Pathophysiological alterations in subthreshold conditions, although not lethal, may induce temporary abnormalities that may be used for targeted therapy as certain antigens or specific proteins may be over-expressed or become more available to portal or systemic drug delivery [38–39]. We aimed to develop an affordable and simple model that allows a versatile setting for experimentation with the live animal while controlling accurately the temperature and timing according to principles of thermal dosimetry [40]. Our experimental heating device offers the possibility of early in vivo modeling of therapeutic approaches combining hyperthermia with pharmacological approaches, thus it is not designed to immediately translate as such in clinical usage. However, we intend to offer this platform as a screening tool, with the possibility to select efficient therapies for translational research in clinical trials.

Delivering microwaves in selected tissue for subthreshold heating in small mammals, comes with problems related to miniaturization and animal protection. Miniaturized antennas using a self-resonant cavity can deliver microwaves with precise frequency [40]. However, a significant local thermal gradient due to unidirectional delivery and possible side irradiation of lateral liver lobes (which we used as controls in normal and metastatic liver), together with the high cost and technical challenges may limit their wide-spread usage. Choosing a microwave oven may appear counterintuitive when aiming to heat a small tissue sample (middle liver lobe) and this approach is generally considered suitable for whole body hyperthermia. While alternative strategies for targeted microwave treatment have been put forth [41], we have shown that efficient screening of the living animal can be achieved and microwave treatment can be restricted to a selected organ without effects on the experimental animal and without safety concerns for the personnel. The effects of thermal gradients within the volume of tissue, such as those generated by the use of antennas [42–44], may be unimportant when thermal destruction is desired, but even small thermal gradients in the range between 40–42°C generated during subthreshold tissue manipulation may alter the outcome of the therapy [26]. Our aim for thermal manipulation is in the same range with HIPEC conditions, in which situation differences in temperature between specific points in the peritoneal cavity can differ by 2° C [45]. For thermal pretreatment, we assume that such a gradient may produce uneven effects that can alter drug delivery if dependent on antigenic or protein expression. In our experiment, we aim to create an overheating of the target tissue of 5°C (above the normal core temperature) and monitoring the temperature in the central part of the experimental lobe ensures a minimal temperature gradient between the core and the surface of the heated liver lobe. Thermal camera readings have major procedure related limitations and were used only to show that the liver surface is adequately heated and no off-target hotspots are created. Accurate 3D temperature dosimetry [46] for such an experimental model is technically difficult, and use of multiple implanted thermocouples is bound to create tissue injury. However, we used electromagnetic/thermal co-simulation that shows a uniform thermal distribution throughout a small target volume (≤2.0cm^3^) as long as the core temperature is maintained at a constant temperature (as recorded by implanted thermocouple). Furthermore, using pulse activation of the magnetron during steady temperature function allows heat to dissipate across the volume producing a more homogenous effect.

Our work is a proof of concept study demonstrating the feasibility of an experimental set-up system for *in vivo* hyperthermia using non-directional microwaves. Targeted heat delivery to specific parts of an organ or specific tissues can be done with our model with the added advantage of very easy adjustment of experimental setup, without additional costs. While not an ideal model for clinical usage for microwave delivery, the proposed system is well adapted for experimental thermal manipulation of living organs in the context of targeted delivery of drugs in heat modified structures. Microwave radiation-induced hyperthermia is emerging as new tool in cancer treatment in adjuvant setting, but is especially susceptible to extraneous energy delivery, affecting off-target tissues [47]. To overcome this limitation, we have used a novel conceptual approach of insulating the entire living organism, with the exception of the targeted tissue. This approach is easy to implement, as the Faraday-type insulator can be conveniently manufactured out of aluminum foil and using a common grounding with the magnetron prevents extraneous electrical discharges and associated hotspots. Furthermore, this insulation technique could be used to target other organs or tissues such as kidneys, intestines, urinary bladder, spleen, and testis.

One desirable feature of our system is the achievement of a rapid increment of tissue temperature with minimal lag between the start of the magnetron and achievement of the desired temperature in the target tissue. In all experiments, the desired temperature was achieved within 7.5 seconds, with minimal variations reflected in the standard deviations of individual measured points. Almost similar intervals are required for each 1°C thermal increment with more variation in individual readings, probably associated with changes in the specific absorption rate, when dielectric tissue properties are changed due to water evaporation. The steep curve suggests that in experimental settings when exposure is in the range of minutes, this activation part can be ignored as far as exposure to a certain temperature needs to be quantified.

Another feature of the system presented here is the stability of the temperature inside target tissue, with minimal variations during the plateau. Setting a desired temperature in the feedback loop is versatile and does not need a set procedure, the system being permanently adapted to local temperature and thus being able to accurately maintain it during the experiment. Taking into account that the system was set to respond to variations of 0.1°C, we consider that the temperatures achieved inside target tissue are very well controlled and the system is functional.

In conclusion, we have developed an inexpensive and easy to manufacture system for targeted hyperthermia using non-directional microwave radiation. This system allows for fine and stable temperature adjustments within the target tissue for various time intervals. Furthermore, the use of non-directional microwave energy is likely to minimize the generation of thermal gradients within the targeted tissue, thus allowing uniform temperature loads.

### Perspectives

Liver tumors may be addressed by heating them at different temperature, but most therapeutic approaches consist in complete destruction using radio, cryo or microwave ablation. Confined tumoral growth can be often destroyed with physical agents, with very good short-term results. This approach is increasingly used, especially in combination with surgery and/ or radio/chemotherapy.

Modern oncology showed that for some non-resectable primary or metastatic tumors, long term management as a chronic disease may produce better results, with better control in its place of occurrence. According to this concept, using bellow-threshold energy delivered in liver tissue, we wish to produce structural changes that will allow further therapeutic manipulations. Using our system, we are capable to induce different degrees of alterations in liver tissue, opening the door for future oncologic therapeutic approaches.

## Supporting information

S1 Fig Virtual instrument hardware setup. J thermocouples (1 and 2) send electric signals that are acquired through analogical ports from NI USB DAQ 6211 acquisition board (6), connected to a computer that runs the software developed under LabView®. The magnetron (4) is automatically controlled through the digital port of the DAQ board (6) that commands the relay (5) to open or close. The microwave trap (3) was installed between the magnetron waveguide (4) and the target in order to provide an additional target.
(TIF)

S2 Fig The virtual instrument (VI) front panel. 1-start button; 2-time interval and temperature limit adjustment; 3-filter accuracy button; 4-recording button; 5-time stamp tag; 6,7-real time temperature readings in analogical and graphic display.
(TIF)

S3 Fig The Filter sub-routine and algorithm. The Filter sub-routine: left side and feed-back algorithm: right side.
(TIF)
