# Supplementary figures and images for: New experimental model for single liver lobe hyperthermia in small animals using non-directional microwaves
Source: PLoS One. 2017 Sep 21;12(9):e0184810. doi: 10.1371/journal.pone.0184810 (PMC5608293; doi:10.1371/journal.pone.0184810)

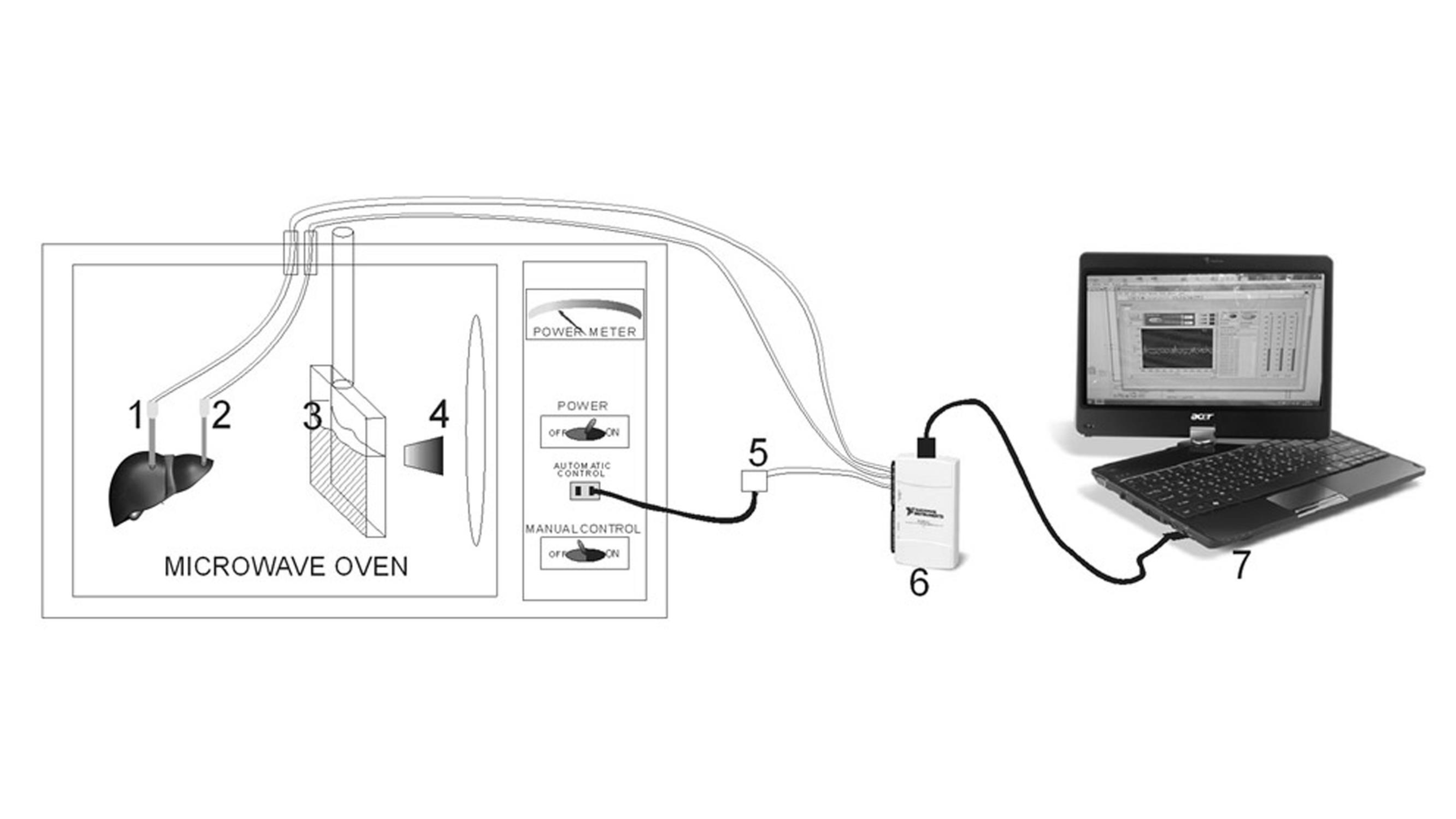

Supplement: S1 Fig — J thermocouples (1 and 2) send electric signals that are acquired through analogical ports from NI USB DAQ 6211 acquisition board (6), connected to a computer that runs the software developed under LabView®. The magnetron (4) is automatically controlled through the digital port of the DAQ board (6) that commands the relay (5) to open or close. The microwave trap (3) was installed between the magnetron waveguide (4) and the target in order to provide an additional target. (TIF) [file pone.0184810.s001.tif]

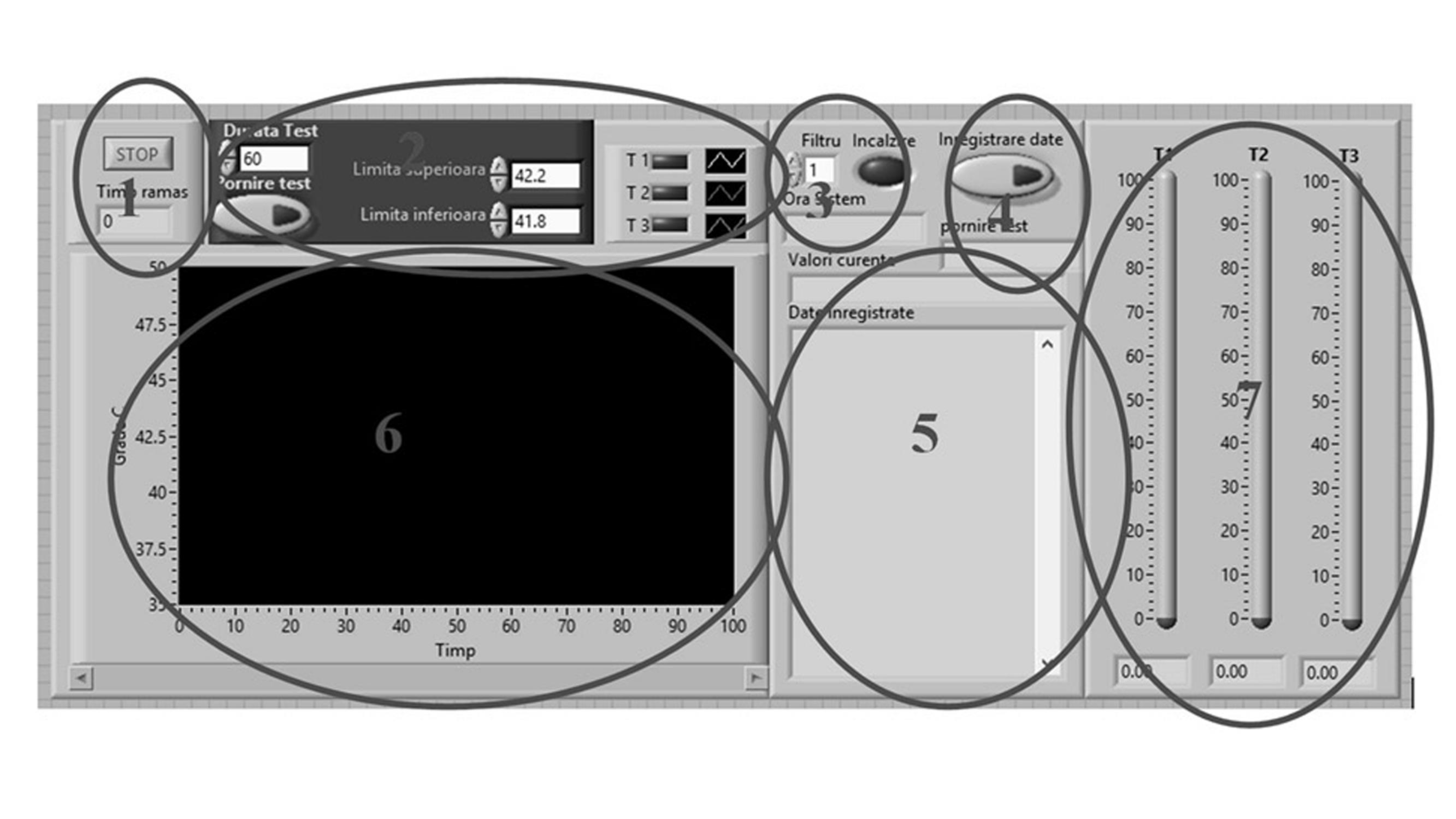

Supplement: S2 Fig — 1-start button; 2-time interval and temperature limit adjustment; 3-filter accuracy button; 4-recording button; 5-time stamp tag; 6,7-real time temperature readings in analogical and graphic display. (TIF) [file pone.0184810.s002.tif]

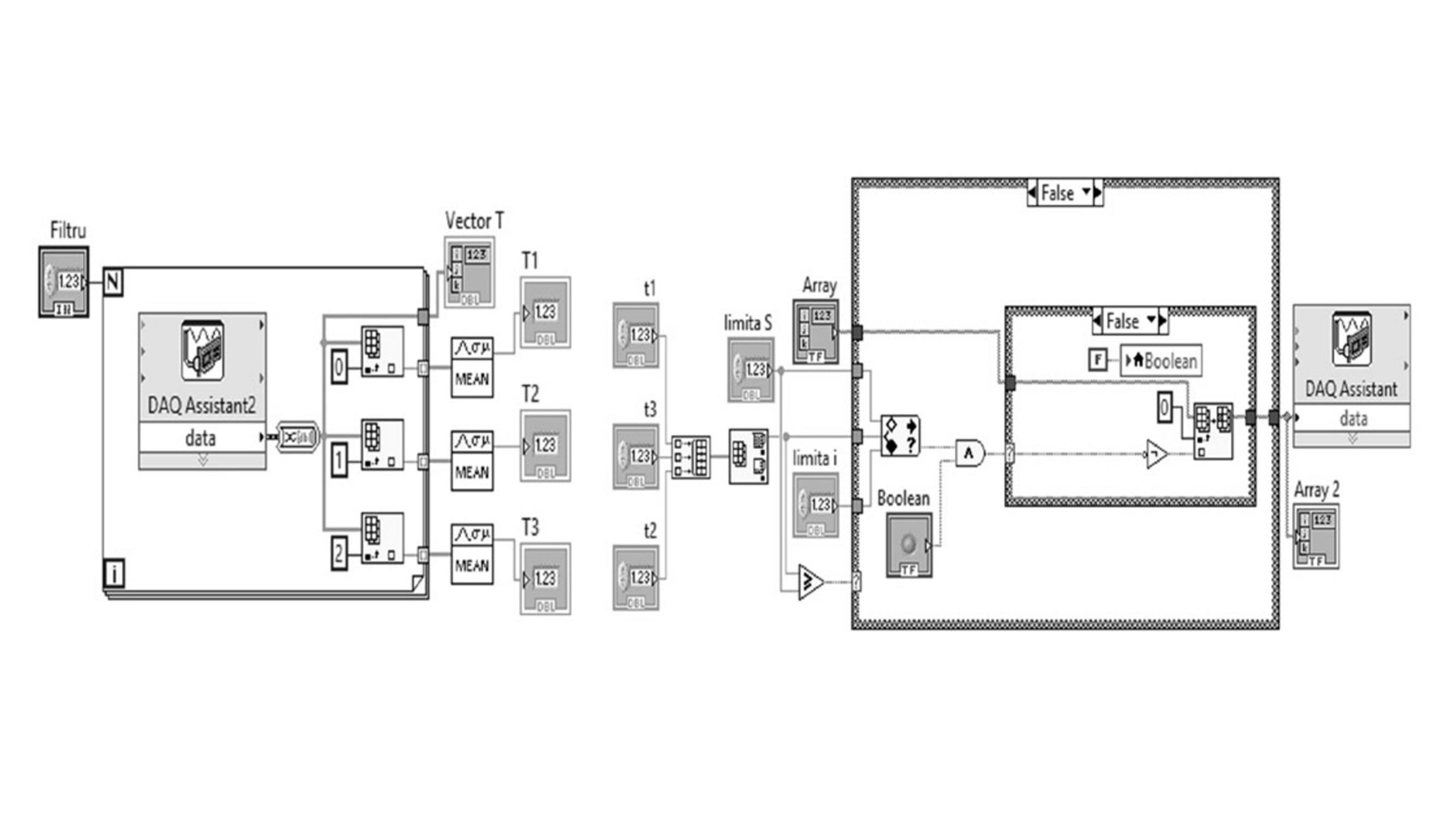

Supplement: S3 Fig — The Filter sub-routine: left side and feed-back algorithm: right side. (TIF) [file pone.0184810.s003.tif]
